# Expanding the BP1-BP2 15q11.2 Microdeletion Phenotype: Tracheoesophageal Fistula and Congenital Cataracts

**DOI:** 10.1155/2013/801094

**Published:** 2013-06-24

**Authors:** D. Wong, S. M. Johnson, D. Young, L. Iwamoto, S. Sood, T. P. Slavin

**Affiliations:** ^1^University of Hawaii John A. Burns School of Medicine, Honolulu, HI 96813, USA; ^2^Department of Surgery, University of Hawaii John A. Burns School of Medicine, Honolulu, HI 96813, USA; ^3^Kapi‘olani Medical Specialists, Kapi‘olani Medical Center for Women and Children, Honolulu, HI 96826, USA; ^4^Department of Pediatrics, University of Hawaii John A. Burns School of Medicine, Honolulu, HI 96813, USA

## Abstract

The proximal q arm of chromosome 15 contains breakpoint regions BP1–BP5 with the classic deletion of BP1–BP3 best known to be associated with Prader-Willi and Angelman syndromes. The region is approximately 500 kb and microdeletions within the BP1-BP2 region have been reported in patients with developmental delay, behavioral abnormalities, and motor apraxia as well as dysmorphic features including hypertelorism, cleft or narrow palate, ear abnormalities, and recurrent upper airway infections. We report two patients with unique, never-before-reported 15q11.2 BP1-2 microdeletion syndrome findings, one with proximal esophageal atresia and distal tracheoesophageal fistula (type C) and one with congenital cataracts. Cataracts have been described in Prader-Willi syndrome but we could not find any description of cataracts in Angelman syndrome. Esophageal atresia and tracheoesophageal fistula have not been reported to our knowledge in either syndrome. A chance exists that both cases are sporadic birth defects; however, the findings of the concomitant microdeletion cannot be overlooked as a possible cause. Based on our review of the literature and the presentation of our patients, we recommend that esophageal atresia and distal tracheoesophageal fistula as well as congenital cataracts be included in the phenotypic spectrum of 15q11.2 BP1-2 microdeletion syndrome.

## 1. Introduction

Breakpoints BP1–BP5 are contained within the proximal q arm of chromosome 15 and the classic deletion of BP1–BP3 in this region is best known to be associated with Prader-Willi syndrome (PWS) and Angelman syndrome (AS) [1]. Specifically, the BP1-BP2 region is composed of approximately 500 kb and contains four nonimprinted genes: *NIPA1*, *NIPA2*, *CYFIP1,* and *TUBGCP5* [2]. These genes are implicated in the compulsive behavior and lower intellectual ability in individuals with Prader-Willi syndrome type I versus type II deletions [3]. Microdeletions within the BP1-BP2 region in 15q11.2 have been previously reported [1]. The emerging phenotype with this microdeletion syndrome is variable and consists of developmental delay, behavioral abnormalities, and motor apraxia as well as dysmorphic features including hypertelorism, cleft or narrow palate, ear abnormalities, and recurrent upper airway infections [4]. Additionally, some patients have little to no symptoms [4]. We report two patients with unique findings, one with proximal esophageal atresia (EA) and distal tracheoesophageal fistula (TEF) (type C) and one with congenital cataracts. These findings were not included in the comprehensive review of nine patients with this microdeletion previously published in this journal [4]. Furthermore, to our knowledge these two findings have never been reported specifically with this microdeletion [4, 5]. We believe these findings should be included in the phenotypic spectrum of this disorder.

Congenital EA can occur with or without TEF, with the most common being type C [6]. TEF/EA can occur independently or in association with other anomalies. The best-known association of TEF/EA is that with vertebral, anal, cardiac, renal, and limb anomalies (VACTERL association). However, no major gene locus for VACTERL association or TEF/EA has been found [7]. Congenital cataracts have a diverse etiology, and, in many children, an underlying cause is not identified [8]. There are multiple genetic loci to which cataracts have been mapped, with the only gene on chromosome 15 being the *CCSSO* gene, which is contained between 15q21 and 15q22 [9].

## 2. Case Report

The first patient was a term female who presented with a proximal EA and distal TEF (type C), patent ductus arteriosus, patent foramen ovale (PFO), and mild pectus excavatum. Prenatal history was significant for polyhydramnios. Family history was negative for similar disorders and no prenatal exposures were noted. Spinal X-ray and abdominal ultrasound were normal at birth. A 263.94 kb deletion was found, del(15)(q11.2q11.2), arr 15q11.2(20,372,901-20,636,841) x1 [UCSC 2006 hg 18 assembly], (RP11-1122J3-), between BP1 and BP2, SignatureChipOS (Spokane, WA, USA). The patient underwent repair of the TEF/EA and has otherwise done well. Clinical examination at four months showed a height of 64.5 cm (90th percentile), weight of 8.30 kg (97th percentile), and head circumference of 43.5 cm (97th percentile). The patient displayed developmentally appropriate behaviors for her age, excellent tone, and aside from mild pectus excavatum, had no other dysmorphic features. 

 The second patient was a term, small for gestational age female, who presented with a small atrial septal defect versus large PFO and bilateral congenital cataracts. She maintained a partial midline maxillary alveolar ridge cleft but was otherwise nondysmorphic. Prenatal history was significant for mild intrauterine growth restriction. Family history was negative for similar disorders and no prenatal exposures were noted. A 477 kb deletion was found, del(15)(q11.2q11.2), arr 15q11.2(22,805,421-23,282,799) [GRCh37/hg 19 assembly], between BP1 and BP2, Affymetrix CytoScan HD platform, Genzyme (Monrovia, CA, USA). Bilateral congenital cataracts were repaired and she is under continued observation for her heart defect. At six months of age, her height was 63.5 cm (25th percentile), weight was 5.9 kg (<3rd percentile), and head circumference was 41 cm (10th percentile). Mild motor delay was noted during a phone followup at ten months of age. She did not sit until around 9 months of age; however, she was beginning to crawl and was able to babble well. She was enrolled in early childhood developmental services.

## 3. Discussion

 Cataracts have been described in PWS but we could not find any description of cataracts in AS [10]. TEF/EA has not been reported to our knowledge in either syndrome. None of the deleted genes are known to be associated with TEF/EA or congenital cataracts. Also, to our knowledge, neither of these findings has been previously reported in 15q11.2 microdeletion syndrome [1, 4, 5]. Heart defects have previously been reported [4, 5]. It should be noted that these two cases do not fit another recognizable genetic syndrome. There is a chance that both cases are sporadic birth defects or that case number one could have VACTERL; however, the findings of the concomitant microdeletion cannot be overlooked as a possible cause. Additionally, the large diversity of dysmorphic findings and organ anomalies in patients with BP1-BP2 deletions supports expanding the phenotypic spectrum [1, 4, 5]. Parental testing was unavailable, but since this deletion has been previously shown to be present in unaffected family members, this would not greatly alter our view that this microdeletion could be causative [1, 4]. Both cases will need close developmental followup as developmental delay and learning disabilities have been frequently described in patients with microdeletions of this region, likely explaining the delays already noted in case two.
